# An empirical research on evaluating banks’ credit assessment of corporate customers

**DOI:** 10.1186/s40064-016-3774-0

**Published:** 2016-12-09

**Authors:** Sang-Bing Tsai, Guodong Li, Chia-Huei Wu, Yuxiang Zheng, Jiangtao Wang

**Affiliations:** 1 School of Economics and Management, Shanghai Maritime University, Shanghai, 201306 China; 2 Law School, Nankai University, Tianjin, 300071 China; 3 Zhongshan Institute, University of Electronic Science and Technology of China, Zhongshan, 528400 Guangdong China; 4 Law School, Nankai University, Tianjin, 300071 China; 5 School of Business, Dalian University of Technology, Panjin, 124221 China; 6 Economics and Management College, Civil Aviation University of China, Tianjin, 300300 China; 7 Institute of Service Industries and Management, Minghsin University of Science Technology, Hsinchu, 304 Taiwan

**Keywords:** Decision-Making Trial and Evaluation Laboratory (DEMATEL), Bank credit, Credit assessment, Decision making, Bank credit risk, Banking supervision law, Management

## Abstract

**Background:**

Under the rapid change of the global financial environment, the risk control of the credit granting is viewed as the foremost task to each bank. With the impact one by one from financial crisis and European debt crisis, the steady bank business is also facing the severe challenge. Banks approve the credits for their customers and then make money from the interest.

**Case presentation:**

Credit granting is not only the primary job but also the main source of income. The quality of credit granting concerns not just the reclaims of creditor’s rights; it also affects the successful running of banks.

**Discussion and Evaluation:**

To enhance the reliability and usefulness of bank credit risk assessment, we first will delve in the facets and indexes in the bank credit risk assessment. Then, we will examine the different dimensions of cause–effect relationships and correlations in the assessment process. Finally, the study focuses on how to raise the functions and benefits of the bank credit risk assessment.

**Conclusions:**

In those five credit risk evaluation dimensions, A “optional capability” and D “competitiveness” are of high relation and high prominence among those dimensions, influencing other items obviously. By actively focusing on these two dimensions and improving their credit risk assessment ability will solve the foremost problems and also solve other facets of credit risk assessment problems at the same time.

## Introduction

The so-called bank credit risk management is through the establishment of credit granting policies, instructions, and coordination between the different sections in the bank, such as the full supervision and control of customers’ credit investigation, choices of payment methods, confirmation of the credit limit, and reclaims of the sum of money, banks are guaranteed to retrieve the receivables back in time safely (Aebi et al. 2012; Benjamin and Charles 2014; Swami 2014).

However, there exists the phenomenon of “credit paradox” in the practice of credit risk management. This so called “credit paradox” is, on one hand, the risk management theory demands banks follow principles of the investment decentralization and diversification in bank credit risk management, to prevent the concentration of the credit authorization. Diversification is even more important and the golden rule to obey since particularly the traditional credit risk management model is lacking the effective credit risk hedge. On the other hand, in real practice, the bank loan business often shows that the diversification principle is not easy to put into practice because many banks do not abide by the diversification rule a lot on their loan business (Berger et al. 2009; Nuno and Manuela 2014; Mora 2014). There are several main reasons to cause “credit paradox” phenomenon, stated blow. (1) For most small–medium sized corporations without credit ratings, the credit situation is reveled by the long-term business partnership between the firms and banks. This way of partnership and information gained tends to make the banks execute loan business with the acquainted business clients. (2) Some banks would limit their loan business companies. Those firms whom the banks are familiar with in certain industry or in certain expertise are banks’ priorities. (3) Diversification of loan business tends to minimize the loan business to small-sized business, unfavorable to attain to the scale of benefits for banks on their loan business. (4) Sometimes the investment on the market would force banks to develop their loan business on certain limited sections or areas.

As to the credit risk assessment of banks, the accurate measurement of the risk is the basic premise. For the reasons stated above, it is extremely difficult to measure the credit risk accurately (Shipra and Yash 2014). So far some credit risk calculation models developed by JP Morgan and other institutes, such as Creditmetrics, CreditRlsk+, KMV models, are still disputable on their effectiveness and reliability. For the time being, there is still lacking effective measurement on credit risk (Aebi et al. 2012; Benjamin and Charles 2014; Shipra and Yash 2014).

Besides, for the related studies about the credit risk index in the past, there are two insufficient points. First, most studies are based on the hypothesis that indexes are independent, with no influences and cause–effect relationships on others. Second, some studies hold the same weight and hypothesis towards the assessment indexes. For solving the insufficiency in the previous studies and upgrading reliability and usefulness of the bank credit risk, this study adopts Decision Making Trial and Evaluation Laboratory (DEMATEL) to develop its theorizing. We first delve in the evaluation facets and indexes in the bank credit risk assessment. Then, we will examine the different dimensions of cause–effect relationships and correlations in the assessment process. Finally, the study focuses on how to raise the functions and benefits of the bank credit risk assessment.

## Literature review

### Bank credit risk and risk management

The credit risk has been the most important management issue to banks. The quality of credit risk management, good or bad, matters a lot to banks which absorb the financial risks in exchange of benefits as their essence of business. The credit risk is like as follows: the borrower or the business counterparties are unable to fulfill the duty of their contracts out of the deterioration and other factors from the entrepreneurs (such as entanglement between firms); therefore this causes the risk of agreement violation and the loss of money. Generally, from different objects and behaviors, the credit risk could be further divided into two types: (1) lending risk, also called, issuer risk. This type of risk is duo to the violation of agreement when borrowers or bond issuers do not repay their debts or their credits get deteriorated, causing the money loss. Lending risk or issuer risk are often correlated to borrowers and bond issuers’ debt credit situations, and correlated to the risk sensitiveness degree of the financial products. (2) The second credit risk is counterparty risk; it could be further divided into two risks: settlement risk and pre-settlement risk. Settlement risk is the risk that counterparties do not fulfill their contract duties in the due settlement time and cause the loss of the equality principal to the bank. Pre-settlement risk is the risk that counterparties violate the agreement before the final settlement day and cause the risk of contract violation to the bank.

The bank credit risk management organizations and functions may appear in different forms. However, the bank should ensure the official positions and related authorities work independently and attributably, not just focusing on the superficial independency, to reach the goal of credit risk management and supervision, such as (Aebi et al. 2012; Jiang and Lo 2014; Nuno and Manuela 2014; Swami 2014):

1. Business functions should be independent from credit granting/verification functions to avoid the interest conflict.
2. Credit verification functions should be independent from credit granting functions to make sure the credit result report objective and just.
3. Accounting functions should be independent from credit granting/verification functions and business functions to avoid fraud and malpractice.
4. The unit responsible for designing, establishing, or executing the credit risk measurement system should be independent from the credit granting functions to keep this unit free of other interruptions.
5. The office worker in charge of verifying the credit risk measurement system should be different from the office worker responsible for designing or choosing the credit risk measurement system to lower the possibility of making errors from the credit risk measurement system.
6. The authorities should obey the regulations to restrict the interested parties in the bank.
7. Re-check the credit granting workers of interest in the bank, such as the credit granting of the general manager and the high-ranked officer.
8. Regularly (at least per year) check the strategies and related policies of the bank credit risk management to confirm that the high-ranked managers carry out the regulations successfully and to make sure the credit granting in accordance with those strategies and related policies. This is then to make the high-ranked managers ultimately responsible for establishing and maintaining the appropriate and effective credit risk management mechanism.
9. Make regular inspection on the bank management information and reflect on the correct credit risk strategies to guarantee the suitability and sufficiency of the bank capital.

### The bank credit risk evaluation methods

For the past 20 years, the development of international bank credit risk management and evaluation has been through the several phases as follows:

1. Influenced by the debt crisis at 1980s, banks mostly began to focus on the preventative measures and management against the credit risk. Thus came out the result of the birth of “Basel Accord” which was a kind of vague analysis of the bank credit risk; through the adopting of different weights on different assets, this agreement quantified the risks.
2. Since 1990s some major banks acknowledged the fact that the credit risk was still the key factor in financial risks and they began to concern about the problems of the credit risk measurement, trying to establish the internal method and model for measuring the credit risk. Among those models, the credit risk management system “Credit Metrics” by J.P. Morgan obtained the widest attention.
3. After the outbreak of Asia financial crisis in 1997, some new phenomenon appeared in the global financial risk. The loss was not necessarily caused by single risk but by the mixture of the credit risk and the market risk etc. Financial crisis motivated people in the banking industry to value the mixture model of the market risk with the credit risk and to focus on the quantification problems of the operation risk. From this phase on, the comprehensive risk management model attained to people’s heed.

Within the traditional credit risk management, the main methods include the Expert System, Internal Ratings Grading Model for Loans, and Z Rating Model. Nevertheless, the modern development of banking makes those methods obsolete and inaccurate. With the advance of modern science and technology and with the enhancement of the management of the market risks plus other risks, modern credit risk management has also been lifted to the certain level. Therefore there appear some credit risk quantification management models such as “Creditmetrics”, “KMV”, “Creditrisk+” models. These models measuring the credit risk still arouse disputes over their effectiveness and reliability. Hence, in all respects, it is still lacking an effective calculating measure to assess the credit risk (Jiang and Lo 2014; Nuno and Manuela 2014; Swami 2014).

According to Dinh and Kleimeier (2007), the determination of loans does not depend on the borrower’s income or the amount of collateral, but rather on the qualitative analysis (of, for example, the borrower’s personality, reputation, or social status). Because the maintenance of social credit relationships is expensive, banks typically adopt the credit scoring model to quantitatively analyze a borrower’s credit situation to determine loans and identify whether a borrower can obey the contract. Banks’ credit assessment of corporate customers is a multiple-criteria decision-making problem in which various elements are comprehensively assessed. The construction of an effective credit assessment model requires that credit staff possess sufficient professional knowledge and practical experience. Previous credit assessment studies have mostly analyzed the opinion of a group of credit staff by using a single precise value, which cannot fully describe the actual distribution of credit staff opinions and tends to diminish minority and peculiar opinions. Therefore, precise values are inapplicable in actual decision environments and constructed credit assessment models do not possess the features of anti-catastrophism and sensitivity, which are the criteria of a superior assessment system (Hsieh 2003). Srinivasan and Kim (1988) stated that credit assessment can be conducted using theory-based scientific and objective methods. The experience of credit decision managers and senior credit staff responsible for credit assessment can be applied to credit assessment models for determining credit categorization and rating weights (Chiou and Shen 2011; Lee et al. 2016).

## Research method

Based on the literature regarding the banks’ approaches and principles of corporate customer credit rating, this study developed five assessment dimensions and 25 criteria, with the definitions listed in Table 1.

**Table 1:** Definitions of assessment dimensions and elements

Dimensions	Criteria	Element definition
A. Operational capability	A1. Operational experience	The operation team possesses abundant operation and management experience
	A2. Industry experience	The operation team has comprehensive experiences regarding industrial growth and decline
	A3. Internal control	Operators have the ability of internally controlling management systems
	A4. Successor system	A system for the succession of managers has been established
	A5. Media management	An enterprise has the ability to employ media communication and to respond to negative coverage
B. Repayment ability	B1. Operation growth	The operational trend and profit demonstrate continued growth
	B2. Fund position	The liquidity fund is sufficient to repay all loans
	B3. Business revenue	Business revenue is derived from the enterprise’s business operation
	B4. Operating revenue	The profit generated from the operation of the enterprise
	B5. Financial planning	The enterprise possesses superior financial dispatch capability to facilitate operational plans
C. Financing capacity	C1. Seasoned equity offering (SEO)	The enterprise is able to support operating funds through SEO
	C2. Bond financing	Corporate bonds are supported by shareholders and investors
	C3. Bank loans	Banks agree to offer loans as the working capital
	C4. Capital turnover	The enterprise has the capital turnover capacity to regulate the source and application of funds
	C5. Capital expenditure	Banks support and provide funds for the enterprise’s long-term capital expenditures
D. Competitive-ness	D1. Product market share	Products have a large market share and superior sales advantage
	D2. Product leading position	Product sales are able to lead and influence market trends
	D3. Price advantage	Product price possesses competitive advantage and price leadership in the market
	D4. Product diversification	Diverse types of products can obtain consumers’ attention
	D5. Product upgrading ability	The enterprise can continue to increase the functions and enhance the performance of products
E. Response ability	E1. Industry cycle	The enterprise can adapt to changes in the industry cycle and improve product features
	E2. Operational crisis	The enterprise is able to respond to and mitigate operational crises
	E3. Ineffective capital turnover	The enterprise has the capacity of capital allocation and turnover for solving financial problems
	E4. Operational strategy	The enterprise can adjust its operational strategies according to changes in the market and industry cycle
	E5. Operational transformation	The enterprise operational style can be transformed according to the economic environment and consumers’ preferences

### DEMATEL model

This study adopted the DEMATEL, which was proposed by Gabus and Fontela who were employed in the Battelle Memorial Institute of Geneva (Gabus and Fontela 1973; Fontela and Gabus 1976; Lee et al. 2014a, b; Guo and Tsai 2015; Guo et al. 2015; Gandhi et al. 2016). At the initial stage, the DEMATEL was used to solve difficult and complex problems such as racial, hunger-related, environmental, and energy-related problems (Hu 2003; Huang 2013; Tsai and Xue 2013; Tsai et al. 2014, 2015, 2016a; Qu et al. 2015). In this study, the DEMATEL was adopted to establish a relationship structure comprising elements used for banks’ credit assessment of corporate customers. When a bank assesses corporate customers, the relationship and degree of influence among the assessment elements are problems common to bank managers. In other words, when a bank manager intends to improve numerous decision-making elements, the optimal approach is to search for the most critical element that influences all other elements.

The DEMATEL structure and calculation steps are summarized and explained in the following sections (Yang and Tzeng 2011; Wu et al. 2013; Liu et al. 2015; Zhang et al. 2015; Tsai 2016; Tsai et al 2016b; Zhou et al. 2016).

The six steps of DEMATEL analysis were implemented in this study:

- Understanding and defining elements

Problems were thoroughly understood, and elements were determined and defined in a complex system through in-depth interviews, a literature review, brainstorming, or the collection of expert opinions.

(b) Determining the correlation among elements and establishing measurement scales

Based on the relationship among elements, a scale of influence degree was developed for pair-wise comparisons. Specifically, each interviewee’s cognition of each aspect’s influence degree was assessed through the pair-wise comparison of aspects (elements). In the assessment scale, 0, 1, 2, 3, and 4 denoted *no influence*, *low influence*, *moderate influence*, *high influence*, and *excessively high influence* among the aspects (elements), respectively.

(c) Constructing a direct-relation matrix

The number of elements was denoted as *n*. Expert opinions were collected by conducting a questionnaire survey. Elements were compared in pairs based on their relationship and degree of influence. Therefore, an *n* × *n* direct-relation matrix (denoted as *X*) was obtained, in which *x*_*ij*_ indicated the influence degree of element *i* on element *j*, and the diagonal elements *x*_*ii*_ were set as 0.

Direct-relation matrix X

1 $$X = \left[ {\begin{array}{*{20}c} 0 & {x_{12} } & \cdots & {x_{1n} } \\ {x_{21} } & 0 & \cdots & {x_{2n} } \\ \vdots & \vdots & \ddots & \vdots \\ {x_{n1} } & {x_{n2} } & \cdots & 0 \\ \end{array} } \right]$$

The symbolic matrix *S* was established, representing the positive and negative influences (denoted as + and −, respectively).

(d) Calculating a normalized direct-relation matrix

2 $${\text{Let}}\;\lambda = \frac{1}{{\mathop {Max}\limits_{1 \le i \le n} \left( {\sum\nolimits_{j = 1}^{n} {x_{ij} } } \right)}}\quad {\text{or}}\quad \lambda = Min\left[ {\frac{1}{{\mathop {Max}\limits_{1 \le i \le n} \left( {\sum\nolimits_{j = 1}^{n} {x_{ij} } } \right)}},\frac{1}{{\mathop {Max}\limits_{1 \le j \le n} \left( {\sum\nolimits_{i = 1}^{n} {x_{ij} } } \right)}}} \right]$$

Through the calculation of Eqs. (2) and (3), the direct-relation matrix was multiplied by *λ* to generate the normalized direct-relation matrix *N*.

3 $$N = \lambda X$$

In addition, DEMATEL analysis assumes that the sum of at least one row of *i* must meet the requirement presented in Eq. (4).

4 $$\sum\limits_{j = 1}^{n} {x_{ij} } < \frac{1}{\lambda }$$

Therefore, the substochastic matrix was computed using the normalized direct-relation matrix *N*.

5 $$\mathop {\lim }\limits_{K \to \infty } N^{K} = O\quad {\text{and}}\quad \mathop {\lim }\limits_{K \to \infty } (I + N + N^{2} + \cdots + N^{K} ) = (I - N)^{ - 1}$$

where *O* represented a null matrix and *I* an identity matrix.

(e) Calculating a direct/indirect relation matrix

When normalized direct-relation matrix *N* met the requirement of Eq. (5), the direct/ indirect relation matrix *T*, also named the total-relation matrix, was obtained using Eq. (6). The indirect relation matrix *H*, also called the total-indirect-relation matrix, was obtained using Eq. (7).

6 $$T = \mathop {\lim }\limits_{K \to \infty } (N + N^{2} + \cdots + N^{K} ) = N(I - N)^{ - 1}$$

7 $$H = \mathop {\lim }\limits_{K \to \infty } (N + N^{2} + \cdots N^{K} ) = N^{2} (I - N)^{\begin{subarray}{l} - 1 \\ \end{subarray} }$$

Let *t*_*ij*_ be the assessment element in the direct/indirect relation matrix *T*, and *i*, $j = 1,2, \ldots ,n$. The sum of rows and that of columns of *T* were calculated using Eqs. (8) and (9). The sum of row *i* was denoted as *D*_*i*_, signifying that the assessment element *i* was the factor that influenced other assessment elements; *R*_*j*_ represented the sum of column *j*, indicating that the assessment element *i* was the result influenced by other assessment elements. Both *D*_*i*_ and *R*_*j*_, which were obtained using the direct/indirect relation matrix *T*, involved direct and indirect influences.

8 $$D_{i} = \sum\limits_{j = 1}^{n} {t_{ij} } \quad (i = 1,2, \ldots n)$$

9 $$R_{i} = \sum\limits_{i = 1}^{n} {t_{ij} } \quad (j = 1,2, \ldots n)$$

(f) Illustrating the causal diagram

In the causal diagram, (*D*_*k*_ + *R*_*k*_, *D*_*k*_ − *R*_*k*_) represented the horizontal and vertical axes. The mean value and 0.0 were used as the dividing points on the horizontal axis (*D*_*k*_ + *R*_*k*_) and vertical axis (*D*_*k*_ − *R*_*k*_), respectively, dividing the causal diagram into four quadrants. The values of (*D*_*k*_ + *R*_*k*_) on the horizontal axis were defined as *prominence*, and $k = i = j = 1,2, \ldots ,n$, indicated the total degree to which an element exerted influence on and was influenced by other elements. Therefore, (*D*_*k*_ + *R*_*k*_) showed the degree to which element *k* was at the core of all problems. In addition, the values of (*D*_*k*_ − *R*_*k*_) on the vertical axis were defined as *relation*, representing the difference in the degree to which an element exerted influence on and was influenced by other elements. Thus, (*D*_*k*_ − *R*_*k*_) showed the causal degree of element *k* in all problems. If the value of (*D*_*k*_ − *R*_*k*_) was positive, the element tended to be a cause; if the value was negative, the element tended to be a result (Hung 2011; Hsu et al. 2013; Ren et al. 2013; Gandhi et al. 2015).

## Results and discussion

### Questionnaires

The five dimensions and 25 elements for banks’ credit assessment of corporate customers were used as items in the DEMATEL expert questionnaire. The questionnaire survey was administered to bank managers in Taiwan. The details are described as follows.

Questionnaires were distributed to 18 Taiwan bank credit managers with more than 20 years of work experience. The DEMATEL questionnaires were distributed between March 16, 2015, and April 30, 2015. The measurement scale was a 5-point scale, with 4 representing maximal influence and 0 representing no influence. The scores between these two values were sequential ratings based on value. The author visited each expert in person, explained the content of the questionnaire, and requested each expert to complete the questionnaire. Overall, 18 questionnaires were distributed and returned. The valid return rate was 100%.

### Results

This study used Matlab software to calculate. The scores from the 18 experts were averaged and rounded to one decimal place to create a table of five criteria, as shown in Table 2.

**Table 2:** Direct-relation matrix *X*

Dimensions	Operational capability (A)	Repayment ability (B)	Financing capacity (C)	Competitiveness (D)	Response ability (E)
Operational capability (A)	0	2.4	2.3	3.4	3.5
Repayment ability (B)	1.2	0	2.5	2	1.5
Financing capacity (C)	1.4	3.2	0	2.1	1.7
Competitiveness (D)	3.5	2.2	2.8	0	2.4
Response ability (E)	1.4	1.6	1.7	1.5	0

Next, the normalized direct-relation matrix was calculated using column vectors and maximums as benchmarks for normalization. The reciprocal of the maximum value within the sum of each column was the λ value. Using Eqs. (2) and (3), the direct-relation matrix X was multiplied by the λ value to obtain the normalized direct-relation matrix N. The influence coefficient was rounded to two decimal places (Table 3).

**Table 3:** Normalized direct-relation matrix *N*

Dimensions	Operational capability (A)	Repayment ability (B)	Financing capacity (C)	Competitiveness (D)	Response ability (E)
Operational capability (A)	0.00	0.21	0.20	0.29	0.30
Repayment ability (B)	0.10	0.00	0.22	0.17	0.13
Financing capacity (C)	0.12	0.28	0.00	0.18	0.15
Competitiveness (D)	0.30	0.19	0.24	0.00	0.21
Response ability (E)	0.12	0.14	0.15	0.13	0.00

Equations (4), (5), (6), (7) were then used to calculate the total-relation matrix T, as shown in Table 4.

**Table 4:** Direct/indirect relation matrix *T*

Dimensions	Operational capability (A)	Repayment ability (B)	Financing capacity (C)	Competitiveness (D)	Response ability (E)
Operational capability (A)	0.55	0.83	0.82	0.86	0.87
Repayment ability (B)	0.46	0.45	0.62	0.56	0.53
Financing capacity (C)	0.51	0.72	0.49	0.62	0.60
Competitiveness (D)	0.77	0.80	0.83	0.62	0.79
Response ability (E)	0.42	0.51	0.51	0.48	0.37

**Fig. 1:**
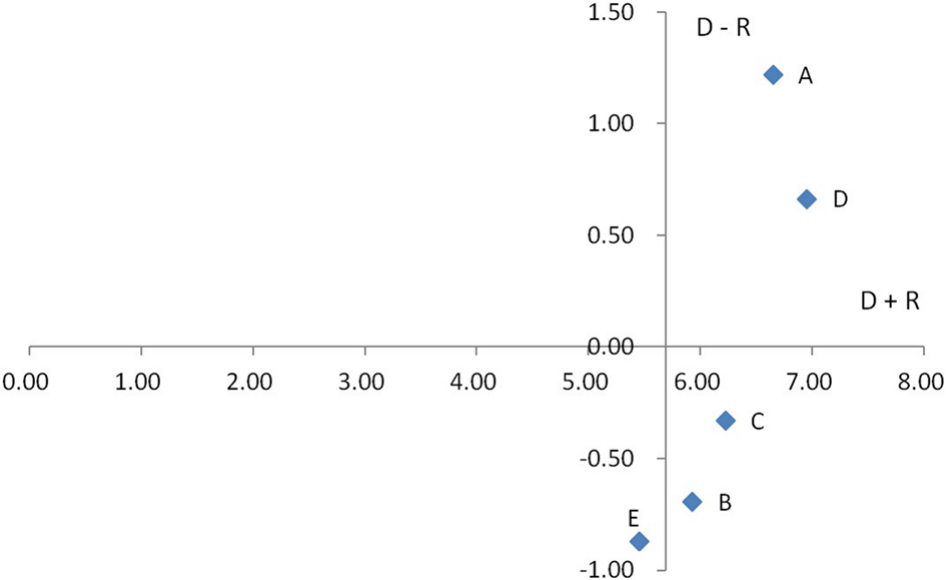
DEMATEL distribution diagram for the five dimensions

Equations (8) and (9) were used to calculate value Di of each column and value Rj of each row to obtain prominence (D + R) and relation (D − R), as shown in Table 4. In addition, the five dimensions were drawn into a figure with prominence as the horizontal axis and relation as the vertical axis, as shown in Fig. 1.

### Discussion

From the results of Table 5 and Fig. 1, the cause–effect relationships and correlations among five evaluation dimensions are interpreted as follows.

**Table 5:** Total direct/indirect influence degree

Dimensions	Sum of rows (D)	Sum of columns (R)	D + R	D − R
Operational capability (A)	3.93	2.71	6.65	1.22
Repayment ability (B)	2.62	3.31	5.92	−0.69
Financing capacity (C)	2.95	3.27	6.22	−0.33
Competitiveness (D)	3.81	3.14	6.95	0.67
Response ability (E)	2.29	3.16	5.46	−0.87
Average			6.24	0.00

1. High relation and high prominence: This category contained A “Operational capability” and D “Competitiveness”. These two dimensions were properties in the cause category and were core influences on the other dimensions. This indicates that these were driving factors and critical problem-solving factors.
2. Low relation and high prominence: This category contained B “Repayment ability” and C “Financing capacity”. These two dimensions were in the effect category and were influenced by the other properties. Although B, C were a property that required improvement, it could not be directly improved because it was in the effect class. Therefore, B, C was relatively irrelevant.
3. Low relation and low prominence: This category contained E “Response ability”. This dimension was influenced by other properties. However, the influences were small. This dimension that these properties were relatively independent.

All in all, in those five credit risk evaluation dimensions above, A “optional capability” and D “competitiveness” are of high relation and high prominence among those dimensions, influencing other items obviously. By actively focusing on these two dimensions and improving their credit risk assessment ability will solve the foremost problems and also solve other facets of credit risk assessment problems at the same time. Thus we suggest the bank corporations pay huge efforts to improve the credit risk assessment and censorship of these two facets, to upgrade the results of the credit risk assessment immediately.

What we will explain is A “optional capability” and D “competitiveness” are two base dimensions for corporation’s competitive ability, competitive advance, and profit gaining ability. If the corporations’ operational capability and competitive ability are the priority to get upgraded, then the corporation’s repayment ability and financing capacity would also arise naturally.

## Conclusion

With the liberalization and globalization of financial development, innovative financial activities flourishing, and the banking business more and more complicated, the financial system risks also gradually increase with time. To effectively adjust to the rapid change of the financial environment, main countries in the world all devote to carrying out financial reforms. Through reflections on financial supervision system and financial regulations, through the improvement of financial credit risk assessment techniques, the banks are urged to sharpen their risk management and corporation administration, to derive a robust financial system and to enhance the country’s financial competitive advantage.

For resolving the insufficiency of the former studies, this study is developed with DEMATEL Model, to increase the reliability and usefulness of the bank credit risk. To enhance the reliability and usefulness of bank credit risk assessment, we first will delve in the facets and indexes in the bank credit risk assessment. Then, we will examine the different dimensions of cause–effect relationships and correlations in the assessment process. Finally, the study focuses on how to raise the functions and benefits of the bank credit risk assessment.

In those five credit risk evaluation dimensions above, A “optional capability” and D “competitiveness” are of high relation and high prominence among those dimensions, influencing other items obviously. By actively focusing on these two dimensions and improving their credit risk assessment ability will solve the foremost problems and also solve other facets of credit risk assessment problems at the same time. Thus we suggest the bank corporations pay huge efforts to improve the credit risk assessment and censorship of these two facets, to upgrade the results of the credit risk assessment immediately.

We suggest the follow-up studies could adopt DEMATEL model and study the cases from other different countries and areas, to discuss the bank credit risk assessment problems and make a comparative study over miscellaneous areas. Other research methods are also recommended to develop other evaluation index system and to make comparisons.
